# Myelin Dynamics Throughout Life: An Ever-Changing Landscape?

**DOI:** 10.3389/fncel.2018.00424

**Published:** 2018-11-19

**Authors:** Jill M. Williamson, David A. Lyons

**Affiliations:** Centre for Discovery Brain Sciences, The University of Edinburgh, Edinburgh, United Kingdom

**Keywords:** myelin, oligodendrocyte, myelin remodelling, circuit plasticity, adaptive myelination

## Abstract

Myelin sheaths speed up impulse propagation along the axons of neurons without the need for increasing axon diameter. Subsequently, myelin (which is made by oligodendrocytes in the central nervous system) allows for highly complex yet compact circuitry. Cognitive processes such as learning require central nervous system plasticity throughout life, and much research has focused on the role of neuronal, in particular synaptic, plasticity as a means of altering circuit function. An increasing body of evidence suggests that myelin may also play a role in circuit plasticity and that myelin may be an adaptable structure which could be altered to regulate experience and learning. However, the precise dynamics of myelination throughout life remain unclear – does the production of new myelin require the differentiation of new oligodendrocytes, and/or can existing myelin be remodelled dynamically over time? Here we review recent evidence for both *de novo* myelination and myelin remodelling from pioneering longitudinal studies of myelin dynamics *in vivo*, and discuss what remains to be done in order to fully understand how dynamic regulation of myelin affects lifelong circuit function.

## Introduction

The human brain undergoes extensive maturation throughout life to facilitate cognitive development. The myelination of axons throughout the nervous system is one such crucial maturation process. In the central nervous system (CNS), glial cells called oligodendrocytes extend many processes into their surrounding environment, which concentrically wrap membrane around axons to form myelin sheaths. Myelin sheaths enable the rapid saltatory conduction of action potentials, by localising voltage-gated Na^+^ channels to short gaps between adjacent sheaths (known as the nodes of Ranvier), and by acting as electrical insulators. Axons that are fully myelinated along their length conduct impulses many times faster than unmyelinated axons of the same cross sectional size (Waxman, 1980). Therefore, myelinated neural circuits conduct information much faster than unmyelinated circuits. Humans are born with a virtually unmyelinated CNS, and the oligodendrocyte population expands dramatically following birth with widespread myelination in the first few years of childhood. Myelination continues through adolescence and into adulthood in a characteristic spatiotemporal manner, correlating with the emergence and maintenance of proper circuit function. For example, the maturation of white matter (the myelin-rich areas of the CNS) is concurrent with development of childhood cognitive processes, such as information processing speed (Mabbott et al., 2006; Scantlebury et al., 2014). Additionally, myelin pathology/abnormalities are seen not only in the demyelinating disease Multiple Sclerosis, but also in several neurodegenerative diseases (Kang et al., 2013; Huang et al., 2015) and neurodevelopmental disorders (Takahashi et al., 2011). However, myelination of individual axons is not an “all-or-nothing” phenomenon. Axons in the CNS exhibit extensive variation in myelin sheath number, sheath length, sheath thickness, and distribution along their length. Many different patterns of myelination exist; for example axons with sparsely myelinated regions have been described in juvenile and adult mouse cortex (Tomassy et al., 2014; Hill et al., 2018; Hughes et al., 2018). Modification to any of these sheath parameters has predictable effects on the conduction speed of the underlying axon – therefore establishing a specific pattern of myelination along an axon may be particularly important to fine-tune circuit function. For instance, axons in the auditory brainstem of gerbils exhibit progressively shorter myelin sheaths along distal regions to ensure the precise timing of signal arrival to facilitate sound localisation (Ford et al., 2015). Subtle changes in the overall pattern of myelination along an axon (either via the addition of new myelin, or the remodelling of existing myelin) could profoundly change the timing of neural impulses in circuits. If myelin is adaptable then modifying such patterns of myelination may represent a powerful mechanism in regulating circuit function throughout life.

Recent evidence suggests that myelin may be adaptable in response to circuit activity. Whole-brain diffusion tensor imaging can be used to measure broad changes in the myelin-rich white matter over time – such experiments in humans and rodents have shown that learning a new task correlates with white matter alterations in relevant brain regions (Scholz et al., 2009; Sampaio-Baptista et al., 2013). Cellular level analyses in animal models demonstrate that the production of new myelinating oligodendrocytes is required for efficient motor learning (McKenzie et al., 2014; Xiao et al., 2016). It is currently hypothesised that neural circuit activity can trigger changes in myelin; an extensive body of research has demonstrated that neuronal activity can influence the proliferation of oligodendrocyte precursor cells (OPCs), the differentiation of oligodendrocytes, and the formation and growth of myelin sheaths. This research, including evidence for the molecular signals involved, has been extensively reviewed elsewhere (Fields, 2015; Almeida and Lyons, 2017; Mount and Monje, 2017). Neuronal activity could drive changes to myelin which could, in turn, change conduction speeds to fine-tune the timings underpinning circuit function.

However, we still do not know whether or how the myelination of circuits is dynamically regulated throughout life. Rodent work indicates that new oligodendrocytes are generated throughout the CNS even in adulthood (Young et al., 2013), and OPCs do reside within the human adult brain (Chang et al., 2000). Carbon-dating analysis of human tissue has identified adult-born oligodendrocytes within the cortex, although the same analyses indicated that the majority of oligodendrocytes in the corpus callosum originate in early childhood (Yeung et al., 2014). However, the neuroimaging studies in humans that correlate white matter structural alterations with task learning suggest that new myelin can be formed throughout life. Such protracted myelination would in principle require lifelong oligodendrocyte production, given that individual myelinating oligodendrocytes have a restricted time window of just a few hours to initiate formation of new sheaths (Watkins et al., 2008; Czopka et al., 2013) and sheath number per oligodendrocyte appears stable over time (Tripathi et al., 2017). One caveat noted by Mount and Monje (2017) is that the “birth” date in the carbon-dating experiment (which identifies the time point of DNA replication during cell division), reflects that of the OPC, not necessarily the differentiated oligodendrocyte. This is important given evidence that OPCs can directly differentiate into oligodendrocytes without cell division, at least in rodents (Hughes et al., 2013). OPCs in the corpus callosum could directly differentiate into oligodendrocytes many years after their terminal cell division; thus the time of differentiation of these new oligodendrocytes cannot be determined by carbon-dating, and so Yeung et al. (2014) may have underestimated the rate of oligodendrocyte production in the adult human brain. We still have much to learn about the relative contributions of oligodendrocyte generation and myelin remodelling to CNS development throughout life.

To fully understand the precise dynamics of oligodendrogenesis, myelin formation and myelin remodelling throughout various stages of life, longitudinal imaging at high-resolution represents the gold-standard approach. Here we provide an overview of recent *in vivo* imaging studies that are beginning to clarify the dynamics of myelination, which will also allow us to begin to understand how such dynamics might impact neural circuit function.

## *De novo* Myelination

**FIGURE 1:**
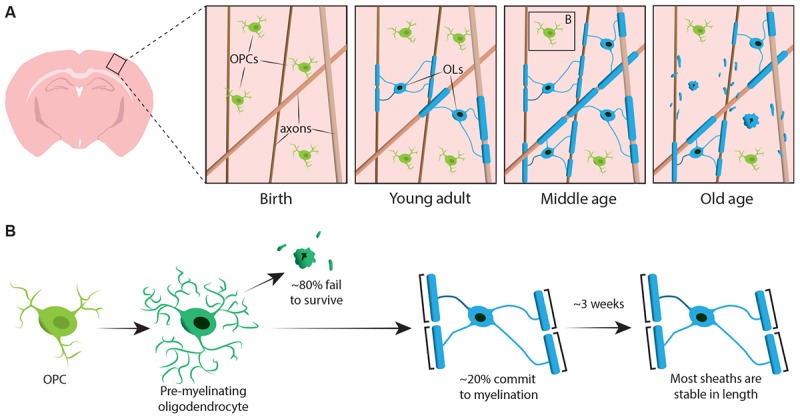
Oligodendrocyte and myelin dynamics in the mammalian cortex throughout life. **(A)** Oligodendrocyte precursor cells (OPCs) continuously generate new myelinating oligodendrocytes (OLs) in the somatosensory cortex from birth up to middle age. The OL population then declines in old age, accompanied by a reduction in myelin coverage. **(B)** Lineage-tracing of single OPCs shows that although premyelinating OLs are continuously produced in adulthood, only approximately 20% survive to myelinate. Most myelin sheaths, once formed, are stable in length over prolonged period of time, indicating that there is normally very little remodelling of existing myelin. Summary of data from Hill et al. (2018) and Hughes et al. (2018).

To begin to definitively address how oligodendrocytes are generated and how myelin is made and dynamically remodelled *in vivo*, two recent studies utilised repeated two-photon imaging of the mouse somatosensory cortex over extended periods of time. Hughes et al. (2018) imaged the cortex of transgenic reporter mice with fluorescently labelled oligodendroglial lineage cells from early adulthood, through middle and old age (approximately P720). They found that the oligodendrocyte population continues to expand and that cortical oligodendrocyte density nearly doubles between young adult and middle-aged stages (Figure 1A). This was accompanied by an over twofold increase in the number of cortical myelin sheaths. But how does oligodendrocyte number increase? In early post-natal development many oligodendrocytes are produced but only a subset survive and go on to myelinate axons (Barres et al., 1992). This appears to be similar in adulthood – by following individual cortical OPCs in the adult cortex for up to 50 days, Hughes et al. (2018) revealed that the majority of newly differentiated oligodendrocytes undergo cell death, with only 22% surviving and committing to myelination (Figure 1B). It remains unknown what proportion of newly differentiated oligodendrocytes are generated following OPC division versus direct differentiation. However, once oligodendrocytes commit to myelination they remain stable, with no evidence of myelinating oligodendrocytes undergoing cell death during a 50-day imaging period.

Similarly, Hill et al. (2018) used transgenic reporters of oligodendrocytes and the label-free spectral confocal reflectance (SCoRe) microscopy technique to image myelin along axons (Schain et al., 2014) in the somatosensory cortex of juvenile, young adult, middle-aged, and old-aged mice (P950). They also found that the oligodendrocyte number continues to expand in adulthood up to P650, and that oligodendrocytes are stable in middle age for up to 80 days of imaging. They found that myelination of the cortex also peaks in middle age at P650, and that oligodendrocyte density significantly falls from its peak (at P650) through very old age (P950) (Figure 1A). This was reflected in a reduction of myelin coverage of layer I cortical axons between P650 and P950. Long-term oligodendrocyte survival may vary between different parts of the CNS. Tripathi et al. (2017) labelled myelinating oligodendrocytes at P60 in mice and then counted how many labelled cells survived until P605 in several CNS regions. They found that in the spinal cord and motor cortex, 60–70% of P60 labelled cells survived, whereas in the corpus callosum, over 90% of P60 labelled cells survived. The reduction in oligodendrocyte number and myelination in certain CNS regions with age raises intriguing questions concerning the role of myelin loss in age-associated cognitive decline. MRI analysis shows that white matter microstructure correlates with fluid intelligence (Ritchie et al., 2015), but also that this white matter microstructure deteriorates with increasing age (Cox et al., 2016). Subsequent age-associated myelin loss could lead to reduced cognitive function due to dysregulation of myelinated circuits.

Could the generation of new oligodendrocytes (and subsequently new myelin) in the adult cortex be responsive to circuit activity? Previous research has shown that reducing sensory input by removing whiskers from mice leads to reduced oligodendrogenesis in the somatosensory cortex (Hill et al., 2014). To investigate this further, Hughes et al. (2018) provided adult (P365) mice with sensory stimulation for 3 weeks by hanging beads in the animal cages to repeatedly stimulate their whiskers and thus the somatosensory cortex. By imaging the somatosensory cortex before and after the 3 weeks, they demonstrated that sensory stimulation increases oligodendrocyte number, potentially due to the increased survival of newly differentiated cells. Kougioumtzidou et al. (2017) provided further evidence that circuit activity may be important in regulating cell survival – they demonstrated that loss of AMPA receptor subunits 2, 3, and 4 in OPCs leads to reduced survival of oligodendrocytes. This suggests that *de novo* myelination could be modulated by cortical circuit activity throughout life, perhaps to fine-tune the function of those same circuits.

Many questions remain to be addressed: what is the effect of oligodendrogenesis and new myelination on actual circuit function? Does neuronal activity enhance the long-term survival of myelinating oligodendrocytes? It is possible that the loss of oligodendrocytes in old age is due to age-associated reduction in neuronal activity, which might, in turn, affect overall oligodendrocyte survival. Alternatively, it may be that oligodendrocytes have a limited lifespan independent of neuronal activity (either intrinsically programmed or influenced by other extrinsic signals associated with aging). In either case, circuit stimulation could help alleviate age-associated myelin loss by either promoting survival of existing oligodendrocytes or stimulating the production of new oligodendrocytes. This in turn could have significant implications in the treatment and prevention of age-associated cognitive decline.

Activity-mediated oligodendrogenesis is not restricted to the somatosensory cortex – young adult mice undergoing motor learning also show an increase in the number of newly differentiated oligodendrocytes in the motor cortex (Xiao et al., 2016). What about other areas of the CNS? Many cortical axons project via the corpus callosum, and therefore, stimulation of cortical circuits could signal to both cortical and callosal OPCs. Two rodent studies have demonstrated that stimulation of cortical neurons induces oligodendrogenesis within the corpus callosum. Gibson et al. (2014) optogenetically stimulated layer V projection neurons in the premotor cortex, finding a rise in OPC proliferation in both the premotor cortex and corpus callosum. This led to an increase in oligodendrocyte number and sheath thickness 4 weeks post-stimulation. More recently, Mitew et al. (2018) used Designer Receptors Exclusively Activated by Designer Drugs to stimulate layer 2/3 somatosensory neurons, and also observed increased OPC proliferation, oligodendrogenesis, and thicker myelin sheaths in the corpus callosum in both juvenile and adult mice. They also demonstrated that new oligodendrocytes preferentially form myelin sheaths on the active axons. This indicates that activity-induced *de novo* myelination can, in principle, target active axons/circuits. It remains unknown how long-lasting changes to myelin in response to neuronal activity might be. The long-term survival of myelinating cells noted by Tripathi et al. (2017), Hill et al. (2018), and Hughes et al. (2018) suggests that once an oligodendrocyte forms myelin sheaths it is likely to survive even if neuronal activity levels return back down to baseline. Whether the myelin sheaths themselves change once neuronal activity returns to normal levels requires more investigation of individual sheath dynamics, which is discussed below.

Thus, it is possible that lifelong *de novo* myelination may occur in many CNS regions, where axons suitable for myelination have sufficient unmyelinated space. However, it remains unclear to what extent oligodendrogenesis continues in different areas of the adult human brain. Carbon-dating analysis suggests that most oligodendrocytes in the corpus callosum tract are generated in early childhood (Yeung et al., 2014). Immunohistochemical analysis of human brain tissue using a novel marker for newly differentiated oligodendrocytes (BCAS1) shows new oligodendrocytes in the frontal cortex even beyond middle-age, but very few new oligodendrocytes in white matter after the third decade of life (Fard et al., 2017). This difference in oligodendrogenesis between species could be a result of scale. Hughes et al.’s (2013) rodent data suggests that oligodendrocytes are generated in huge excess, with continuous pruning of almost 80% of cells. Given the energy cost of such a process, is this mechanism sustainable throughout life in an organ the size of the human brain? Perhaps in the human brain there is limited oligodendrocyte overproduction, because of a need for more protracted myelination of the larger CNS, or because signals such as neuronal activity stimulate OPCs to differentiate into oligodendrocytes as and when required.

## Myelin Remodelling

The remodelling of existing myelin sheaths could alter conduction properties without the need for generating new oligodendrocytes or myelin. Changing the lengths of existing myelin sheaths could change the myelin coverage along an axon and the distance between nodes of Ranvier (which would both impact conduction speeds). In addition, even very subtle myelin remodelling could alter the lengths of the nodes themselves. It has recently been shown that node length can vary extensively in the optic nerve and in the cortex, and that changing the node lengths along an axon can, in principle, also significantly alter conduction velocity (Arancibia-Cárcamo et al., 2017). Whether changes to node of Ranvier are primarily driven by myelination or reorganisation of the axon itself remains to be determined.

Both Hill et al. (2018) and Hughes et al. (2018) performed longitudinal study of individual myelin sheaths in the mouse somatosensory cortex for several weeks to assess if sheath lengths are dynamically regulated. Hill et al. (2018) found that, in early adulthood (P90–120), although some sheaths exhibit extension or shrinkage, 81% of sheaths observed were stable. More sheaths may become stable in length with age; Hughes et al. (2018) followed sheaths in older (P365) animals and saw that 99% of sheaths remained stable over 3 weeks (Figure 1B).

Similar sheath length stability has also been described elsewhere; Auer et al. (2018) used larval zebrafish to investigate whether individual sheaths can change in length over time by performing time-course live imaging of fluorescently labelled myelin sheaths. They found that individual sheaths undergo rapid but variable growth in the first few days after formation, before stabilising their sheath lengths. Once stabilised, sheaths only continue to grow to accommodate the overall growth of the animal.

Why do some sheaths in the cortex change in length, whilst others do not? This may reflect diversity in the demands of distinct neural circuits. Axonal diversity has been observed during initial myelination in the zebrafish spinal cord, where some axons use synaptic vesicle release to regulate myelin sheath number and length while others do not (Koudelka et al., 2016). This raises the intriguing hypothesis that only some axons are capable of regulating myelin via activity-related signals. Hughes et al. (2018) found that their sensory stimulation paradigm did not increase the proportion of dynamic sheaths in the somatosensory cortex. However, more detailed analysis of axon subtype diversity coupled with longitudinal study of sheath length dynamics could confirm if sheath length remodelling is specific to certain circuits.

**FIGURE 2:**
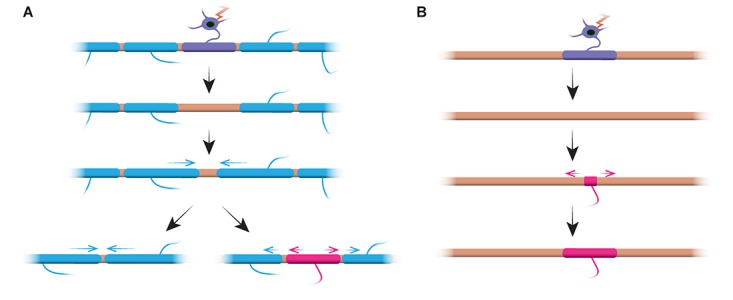
Myelin remodelling can occur *in vivo*. **(A)** Ablation of single sheaths on a fully myelinated axon can induce the rapid growth of neighbouring sheaths to cover the gap. This gap can either be covered entirely by the neighbouring sheaths, or the original myelination profile can be restored by the addition of a new sheath. **(B)** Ablation of a sheath on a sparsely myelinated axon is followed by formation of a new myelin sheath of identical size and location to the ablated predecessor sheath. Summary of data from Auer et al. (2018).

Does sheath length stability reflect an inability of sheaths to remodel? Experiments in the zebrafish suggest that sheath length remodelling can be induced when the myelination profile of an axon is disrupted. Auer et al. (2018) ablated single oligodendrocytes and therefore sparsely removed sheaths along axons. They found that when a single myelin sheath is lost on a fully myelinated axon, the neighbouring sheaths could reinitiate rapid growth to cover the unmyelinated gap. In several cases a new myelin sheath would form in place of its predecessor and could even push back against the invading neighbour sheaths to restore the original pattern of myelination (Figure 2A). Therefore, sometimes a specific myelination pattern is preferentially maintained, even after myelin disruption. This may be to sustain the optimised conduction properties of the underlying axon. Auer et al. (2018) observed sparsely myelinated axons in the larval zebrafish, as previously identified in the rodent cortex. Interestingly, they found that upon ablation of single sheaths on such sparsely myelinated axons a new sheath formed in virtually the same place as the ablated sheath, even along an otherwise unmyelinated stretch of the axon (Figure 2B). Thus the myelination patterns along sparsely myelinated axons also appear to be stably maintained in zebrafish, as suggested by Hill et al. (2018) in rodents. The function of sparse myelination profiles remains unknown. Such patterns may allow for more dynamic fine-tuning of single axon function over time, although it is also possible that such unmyelinated gaps may facilitate gradual myelination to maintain consistent conduction times within circuits, as the animal grows and/or axon lengths change.

Do stable myelin sheaths in mammals also have this capacity to remodel when the myelination pattern is disrupted? Further longitudinal studies coupled with demyelination are required to answer this question. It is possible that such remodelling is not induced by neuronal activity but is a compensatory mechanism for myelin loss. Age-associated oligodendrocyte loss could trigger remodelling of surviving sheaths to cover denuded portions of axon and therefore help maintain circuit function. Live imaging of myelin sheaths in old age could determine if this is the case.

The live imaging studies discussed here have all assessed myelin sheath length dynamics, but not myelin sheath thickness. Can sheath thickness be dynamically regulated? Stimulating PI3K/AKT/mTOR signalling in oligodendrocytes of adult mice triggers additional myelin wrapping to increase sheath thickness (Snaidero et al., 2014). This may be modulated by circuit activity, as neuronal stimulation leads to increased sheath thickness in both juvenile and adult mice (Gibson et al., 2014; Mitew et al., 2018). This highlights the need to image all sheath parameters longitudinally to fully understand the dynamics of sheath remodelling. There is a need for live-imaging modalities to accurately measure sheath thickness along axons, as currently this requires cross-sectional measurement via electron microscopy, which limits analysis to a single time-point. Some label-free imaging techniques, such as third harmonic generation microscopy and spectral reflectometry, show promise for performing such measurements (Lim et al., 2014; Kwon et al., 2017). Coupling these techniques with longitudinal studies of the rodent cortex could determine whether established myelin sheaths can adjust their thickness, or if neuronal activity simply pushes *de novo* myelination to produce thicker sheaths.

It therefore seems that, although myelin sheaths are capable of remodelling when myelin is disrupted, most sheaths are generally stable in length. This stability is potentially due to the maintenance of early established myelination patterns optimised for circuit function.

## The Future

Recent mammalian imaging studies have focused on *de novo* myelination and sheath remodelling in cortical grey matter. Cortical circuits receive and send information via many regions, such as the spinal cord and corpus callosum, and so changes to myelin in several different CNS areas could alter signalling in a single circuit. The CNS is traditionally described by appearance after formaldehyde fixation, where “white matter” describes the heavily myelinated axonal tracts, while “grey matter” describes regions densely packed with neuronal cell bodies, dendrites, and synapses. However, this classification is overly simplistic; OPCs produce myelinating oligodendrocytes in both grey and white matter (Dawson et al., 2003), and in fact there is emerging evidence of diversity in the oligodendroglial lineage and in patterns of myelination in both grey and white matter (Rivers et al., 2008; Viganò et al., 2013; Young et al., 2013; Bechler et al., 2015). Such diversity may reflect the unique requirements of myelin in distinct areas, and potentially on distinct circuits, of the CNS. Further longitudinal imaging studies are required to better understand the dynamics of *de novo* myelination and sheath remodelling in areas of the CNS beyond the cortex.

While the optical transparency of the larval zebrafish lends itself to non-invasive live imaging, performing such experiments in the mammalian CNS is more invasive and technically challenging. Hill et al. (2018) and Hughes et al. (2018) utilised two-photon microscopy with cranial imaging windows to image depths up to 400 μm into the cortex. Similar techniques could be used to image superficial myelinated tracts in the spinal cord over time (Locatelli et al., 2018) but deeper CNS regions cannot be penetrated by two-photon microscopy alone. One alternative is to use two-photon microendoscopy, where a microendoscope probe with a gradient refractive index (GRIN) lens is inserted into the tissue to image cells deeper in the brain [previously used to image CA1 neurons of the hippocampus (Jung et al., 2004; Levene et al., 2004)]. However, endoscope insertion may lead to inflammatory responses which could impact myelination. An alternative could be three-photon microscopy using the cranial imaging window method, which has also been previously used to image the hippocampus (Horton et al., 2013; Ouzounov et al., 2017). Three-photon microscopy gives a significantly greater signal-to-background ratio than two-photon microscopy and can therefore be used to image deeper tissue structures.

It is particularly important to consider not only different CNS regions, but different neurons within these regions. Previous research suggests that there are mechanistic differences in how distinct neuron subtypes regulate their myelination (Koudelka et al., 2016). Additionally, there may be diversity in local regulation of myelin. It is essential to remember that different parts of the CNS are not separate entities but are interconnected. Integrating mesoscale connectomics, which focuses on understanding the connections of different neuron subtypes across different regions (Zeng, 2018), will be crucial to our understanding of how lifelong myelination dynamics vary between different circuits.

What is the functional consequence of myelin regulation along distinct circuits? Thus far, the functional implications can only be inferred by correlations with behaviour. Ultimately, there is a need to couple measurement of myelin dynamics with direct assessment of circuit activity. This will require the recording of neuronal activity during longitudinal studies of myelination in order to directly connect *de novo* myelination or sheath remodelling observed to changes in circuit function with time. It will be important to measure the myelin dynamics and electrophysiological activity of individual neurons and axons to determine how changes in the various myelin sheath parameters actually affects the conduction properties at the single cell level, as well as assessing activity on a population level. Tools such as genetically encoded Ca^2+^ or voltage indicators allow relatively non-invasive recording of circuit activity, and can even be used to assess whole-brain circuit activity (Ahrens et al., 2012; Lovett-Barron et al., 2017).

## Conclusion

The myelination of axons represents a powerful potential mechanism to regulate circuit function throughout life. Research has demonstrated that *de novo* myelination in the cortex (via the production of new oligodendrocytes) occurs even in adulthood, and that this can be enhanced by stimulating circuit activity. Once myelin has formed, it is stable with little turnover of oligodendrocytes and limited remodelling of the lengths of existing myelin sheaths. However, these stable structures may retain the capacity to remodel if myelin is disturbed. This has interesting implications concerning the plasticity of myelin in maintaining circuit function during injury, disease, and old age. Precisely how changes in myelination affect the function of the underlying circuit remains to be seen. Ultimately, a circuit-level approach, integrating analysis of myelin dynamics with direct measurement of circuit function, is required to fully appreciate how dynamic myelination influences overall nervous system function throughout life.

## Author Contributions

All authors listed have made a substantial, direct and intellectual contribution to the work, and approved it for publication.

## Conflict of Interest Statement

The authors declare that the research was conducted in the absence of any commercial or financial relationships that could be construed as a potential conflict of interest.

## References

[B1] Ahrens M. B. Li J. M. Orger M. B. Robson D. N. Schier A. F. Engert F. (2012). Brain-wide neuronal dynamics during motor adaptation in zebrafish. *Nature* 485 471–477. 10.1038/nature11057 22622571 PMC3618960

[B2] Almeida R. G. Lyons D. A. (2017). On myelinated axon plasticity and neuronal circuit formation and function. *J. Neurosci.* 37 10023–10034. 10.1523/JNEUROSCI.3185-16.2017 29046438 PMC6596541

[B3] Arancibia-Cárcamo I. L. Ford M. C. Cossell L. Ishida K. Tohyama K. Attwell D. (2017). Node of Ranvier length as a potential regulator of myelinated axon conduction speed. *eLife* 6:e23329. 10.7554/eLife.23329 28130923 PMC5313058

[B4] Auer F. Vagionitis S. Czopka T. (2018). Evidence for myelin sheath remodeling in the CNS revealed by *in vivo* imaging. *Curr. Biol.* 28 549–559. 10.1016/j.cub.2018.01.017 29429620

[B5] Barres B. A. Hart I. K. Coles H. S. R. Burne J. F. Voyvodic J. T. Richardson W. D. (1992). Cell death and control of cell survival in the oligodendrocyte lineage. *Cell* 70 31–46. 10.1016/0092-8674(92)90531-G 1623522

[B6] Bechler M. E. Byrne L. Ffrench-Constant C. (2015). CNS myelin sheath lengths are an intrinsic property of oligodendrocytes. *Curr. Biol.* 25 2411–2416. 10.1016/j.cub.2015.07.056 26320951 PMC4580335

[B7] Chang A. Nishiyama A. Peterson J. Prineas J. Trapp B. D. (2000). NG2-positive oligodendrocyte progenitor cells in adult human brain and multiple sclerosis lesions. *J. Neurosci.* 20 6404–6412. 10.1523/JNEUROSCI.20-17-06404.2000 10964946 PMC6772992

[B8] Cox S. R. Ritchie S. J. Tucker-Drob E. M. Liewald D. C. Hagenaars S. P. Davies G. (2016). Ageing and brain white matter structure in 3,513 UK Biobank participants. *Nat. Commun.* 7:13629. 10.1038/ncomms13629 27976682 PMC5172385

[B9] Czopka T. Ffrench-Constant C. Lyons D. A. (2013). Individual oligodendrocytes have only a few hours in which to generate new myelin sheaths *in vivo*. *Dev. Cell* 25 599–609. 10.1016/j.devcel.2013.05.013 23806617 PMC4013507

[B10] Dawson M. R. L. Polito A. Levine J. M. Reynolds R. (2003). NG2-expressing glial progenitor cells: an abundant and widespread population of cycling cells in the adult rat CNS. *Mol. Cell. Neurosci.* 24 476–488. 10.1016/S1044-7431(03)00210-0 14572468

[B11] Fard M. K. Van der Meer F. Sánchez P. Cantuti-Castelvetri L. Mandad S. Jäkel S. (2017). BCAS1 expression defines a population of early myelinating oligodendrocytes in multiple sclerosis lesions. *Sci. Transl. Med.* 9:eaam7816. 10.1126/scitranslmed.aam7816 29212715 PMC7116798

[B12] Fields R. D. (2015). A new mechanism of nervous system plasticity: activity-dependent myelination. *Nat. Rev. Neurosci.* 16 756–767. 10.1038/nrn4023 26585800 PMC6310485

[B13] Ford M. C. Alexandrova O. Cossell L. Stange-Marten A. Sinclair J. Kopp-Scheinpflug C. (2015). Tuning of Ranvier node and internode properties in myelinated axons to adjust action potential timing. *Nat. Commun.* 6:8073. 10.1038/ncomms9073 26305015 PMC4560803

[B14] Gibson E. M. Purger D. Mount C. W. Goldstein A. K. Lin G. L. Wood L. S. (2014). Neuronal activity promotes oligodendrogenesis and adaptive myelination in the mammalian brain. *Science* 344:1252304. 10.1126/science.1252304 24727982 PMC4096908

[B15] Hill R. A. Li A. M. Grutzendler J. (2018). Lifelong cortical myelin plasticity and age-related degeneration in the live mammalian brain. *Nat. Neurosci.* 21 683–695. 10.1038/s41593-018-0120-6 29556031 PMC5920745

[B16] Hill R. A. Patel K. D. Goncalves C. M. Grutzendler J. Nishiyama A. (2014). Modulation of oligodendrocyte generation during a critical temporal window after NG2 cell division. *Nat. Neurosci.* 17 1518–1527. 10.1038/nn.3815 25262495 PMC4275302

[B17] Horton N. G. Wang K. Kobat D. Clark C. G. Wise F. W. Schaffer C. B. (2013). *In vivo* three-photon microscopy of subcortical structures within an intact mouse brain. *Nat. Photonics* 7 205–209. 10.1038/NPHOTON.2012.336 24353743 PMC3864872

[B18] Huang B. Wei W. Wang G. Gaertig M. A. Feng Y. Wang W. (2015). Mutant huntingtin downregulates myelin regulatory factor-mediated myelin gene expression and affects mature oligodendrocytes. *Neuron* 85 1212–1226. 10.1016/j.neuron.2015.02.026 25789755 PMC4366619

[B19] Hughes E. G. Kang S. H. Fukaya M. Bergles D. E. (2013). Oligodendrocyte progenitors balance growth with self-repulsion to achieve homeostasis in the adult brain. *Nat. Neurosci.* 16 668–676. 10.1038/nn.3390 23624515 PMC3807738

[B20] Hughes E. G. Orthmann-Murphy J. L. Langseth A. J. Bergles D. E. (2018). Myelin remodeling through experience-dependent oligodendrogenesis in the adult somatosensory cortex. *Nat. Neurosci.* 21 696–706. 10.1038/s41593-018-0121-5 29556025 PMC5920726

[B21] Jung J. C. Mehta A. D. Aksay E. Stepnoski R. Schnitzer M. J. (2004). *In vivo* mammalian brain imaging using one- and two-photon fluorescence microendoscopy. *J. Neurophysiol.* 92 3121–3133. 10.1152/jn.00234.2004 15128753 PMC2826362

[B22] Kang S. H. Li Y. Fukaya M. Lorenzini I. Cleveland D. W. Ostrow L. W. (2013). Degeneration and impaired regeneration of gray matter oligodendrocytes in amyotrophic lateral sclerosis. *Nat. Neurosci.* 16 571–579. 10.1038/nn.3357 23542689 PMC3637847

[B23] Koudelka S. Voas M. G. Almeida R. G. Baraban M. Soetaert J. Meyer M. P. (2016). Individual neuronal subtypes exhibit diversity in CNS myelination mediated by synaptic vesicle release. *Curr. Biol.* 26 1447–1455. 10.1016/j.cub.2016.03.070 27161502 PMC4906267

[B24] Kougioumtzidou E. Shimizu T. Hamilton N. B. Tohyama K. Sprengel R. Monyer H. (2017). Signalling through AMPA receptors on oligodendrocyte precursors promotes myelination by enhancing oligodendrocyte survival. *eLife* 6:e28080. 10.7554/eLife.28080 28608780 PMC5484614

[B25] Kwon J. Kim M. Park H. Kang B.-M. Jo Y. Kim J.-H. (2017). Label-free nanoscale optical metrology on myelinated axons *in vivo*. *Nat. Commun.* 8:1832. 10.1038/s41467-017-01979-2 29184114 PMC5705720

[B26] Levene M. J. Dombeck D. A. Kasischke K. A. Molloy R. P. Webb W. W. (2004). *In vivo* multiphoton microscopy of deep brain tissue. *J. Neurophysiol.* 91 1908–1912. 10.1152/jn.01007.2003 14668300

[B27] Lim H. Sharoukhov D. Kassim I. Zhang Y. Salzer J. L. Melendez-Vasquez C. V. (2014). Label-free imaging of Schwann cell myelination by third harmonic generation microscopy. *Proc. Natl. Acad. Sci. U.S.A.* 111 18025–18030. 10.1073/pnas.1417820111 25453108 PMC4273419

[B28] Locatelli G. Theodorou D. Kendirli A. Jordão M. J. C. Staszewski O. Phulphagar K. (2018). Mononuclear phagocytes locally specify and adapt their phenotype in a multiple sclerosis model. *Nat. Neurosci.* 21 1196–1208. 10.1038/s41593-018-0212-3 30127427

[B29] Lovett-Barron M. Andalman A. S. Allen W. E. Vesuna S. Kauvar I. Burns V. M. (2017). Ancestral circuits for the coordinated modulation of brain state. *Cell* 171 1411–1423. 10.1016/j.cell.2017.10.021 29103613 PMC5725395

[B30] Mabbott D. J. Noseworthy M. Bouffet E. Laughlin S. Rockel C. (2006). White matter growth as a mechanism of cognitive development in children. *Neuroimage* 33 936–946. 10.1016/j.neuroimage.2006.07.024 16978884

[B31] McKenzie I. A. Ohayon D. Li H. Paes de Faria J. Emery B. Tohyama K. (2014). Motor skill learning requires active central myelination. *Science* 346 318–322. 10.1126/science.1254960 25324381 PMC6324726

[B32] Mitew S. Gobius I. Fenlon L. R. McDougall S. J. Hawkes D. Xing Y. L. (2018). Pharmacogenetic stimulation of neuronal activity increases myelination in an axon-specific manner. *Nat. Commun.* 9:306. 10.1038/s41467-017-02719-2 29358753 PMC5778130

[B33] Mount C. W. Monje M. (2017). Wrapped to adapt: experience-dependent myelination. *Neuron* 95 743–756. 10.1016/j.neuron.2017.07.009 28817797 PMC5667660

[B34] Ouzounov D. G. Wang T. Wang M. Feng D. D. Horton N. G. Cruz-Hernández J. C. (2017). *In vivo* three-photon imaging of activity of GCaMP6-labeled neurons deep in intact mouse brain. *Nat. Methods* 14 388–390. 10.1038/nmeth.4183 28218900 PMC6441362

[B35] Ritchie S. J. Bastin M. E. Tucker-Drob E. M. Maniega S. M. Engelhardt L. E. Cox S. R. (2015). Coupled changes in brain white matter microstructure and fluid intelligence in later life. *J. Neurosci.* 35 8672–8682. 10.1523/JNEUROSCI.0862-15.2015 26041932 PMC4452562

[B36] Rivers L. E. Young K. M. Rizzi M. Jamen F. Psachoulia K. Wade A. (2008). PDGFRA/NG2 glia generate myelinating oligodendrocytes and piriform projection neurons in adult mice. *Nat. Neurosci.* 11 1392–1401. 10.1038/nn.2220 18849983 PMC3842596

[B37] Sampaio-Baptista C. Khrapitchev A. A. Foxley S. Schlagheck T. Scholz J. Jbabdi S. (2013). Motor skill learning induces changes in white matter microstructure and myelination. *J. Neurosci.* 33 19499–19503. 10.1523/jneurosci.3048-13.2013 24336716 PMC3858622

[B38] Scantlebury N. Cunningham T. Dockstader C. Laughlin S. Gaetz W. Rockel C. (2014). Relations between white matter maturation and reaction time in childhood. *J. Int. Neurophsychol. Soc.* 20 99–112. 10.1017/S1355617713001148 24168858

[B39] Schain A. J. Hill R. A. Grutzendler J. (2014). Label-free *in vivo* imaging of myelinated axons in health and disease with spectral confocal reflectance microscopy. *Nat. Med.* 20 443–449. 10.1038/nm.3495 24681598 PMC3981936

[B40] Scholz J. Klein M. C. Behrens T. E. J. Johansen-berg H. (2009). Training induces changes in white-matter architecture. *Nat. Neurosci.* 12 1370–1371. 10.1038/nn.2412 19820707 PMC2770457

[B41] Snaidero N. Möbius W. Czopka T. Hekking L. H. P. Mathisen C. Verkleij D. (2014). Myelin membrane wrapping of CNS axons by PI(3,4,5)P3-dependent polarized growth at the inner tongue. *Cell* 156 277–290. 10.1016/j.cell.2013.11.044 24439382 PMC4862569

[B42] Takahashi N. Sakurai T. Davis K. L. Buxbaum J. D. (2011). Linking oligodendrocyte and myelin dysfunction to neurocircuitry abnormalities in schizophrenia. *Prog. Neurobiol.* 93 13–24. 10.1016/j.pneurobio.2010.09.004 20950668 PMC3622281

[B43] Tomassy G. S. Berger D. R. Chen H.-H. Kasthuri N. Hayworth K. J. Vercelli A. (2014). Distinct profiles of myelin distribution along single axons of pyramidal neurons in the neocortex. *Science* 344 319–324. 10.1126/science.1249766 24744380 PMC4122120

[B44] Tripathi R. B. Jackiewicz M. Mckenzie I. A. Kougioumtzidou E. Grist M. Richardson W. D. (2017). Remarkable stability of myelinating oligodendrocytes in mice. *Cell Rep.* 21 316–323. 10.1016/j.celrep.2017.09.050 29020619 PMC5643547

[B45] Viganò F. Möbius W. Götz M. Dimou L. (2013). Transplantation reveals regional differences in oligodendrocyte differentiation in the adult brain. *Nat. Neurosci.* 16 1370–1372. 10.1038/nn.3503 23995069

[B46] Watkins T. A. Emery B. Mulinyawe S. Barres B. A. (2008). Distinct stages of myelination regulated by gamma-secretase and astrocytes in a rapidly myelinating CNS coculture system. *Neuron* 60 555–569. 10.1016/j.neuron.2008.09.011 19038214 PMC2650711

[B47] Waxman S. G. (1980). Determinants of conduction velocity in myelinated nerve fibers. *Muscle Nerve* 3 141–150. 10.1002/mus.880030207 6245357

[B48] Xiao L. Ohayon D. Mckenzie I. A. Sinclair-Wilson A. Wright J. L. Fudge A. D. (2016). Rapid production of new oligodendrocytes is required in the earliest stages of motor-skill learning. *Nat. Neurosci.* 19 1210–1217. 10.1038/nn.4351 27455109 PMC5008443

[B49] Yeung M. S. Y. Zdunek S. Bergmann O. Bernard S. Salehpour M. Alkass K. (2014). Dynamics of oligodendrocyte generation and myelination in the human brain. *Cell* 159 766–774. 10.1016/j.cell.2014.10.011 25417154

[B50] Young K. M. Psachoulia K. Tripathi R. B. Dunn S.-J. Cossell L. Attwell D. (2013). Oligodendrocyte dynamics in the healthy adult CNS: evidence for myelin remodeling. *Neuron* 77 873–885. 10.1016/j.neuron.2013.01.006 23473318 PMC3842597

[B51] Zeng H. (2018). Mesoscale connectomics. *Curr. Opin. Neurobiol.* 50 154–162. 10.1016/j.conb.2018.03.003 29579713 PMC6027632

